# Effect of Low-Temperature Al_2_O_3_ ALD Coating on Ni-Rich Layered Oxide Composite Cathode on the Long-Term Cycling Performance of Lithium-Ion Batteries

**DOI:** 10.1038/s41598-019-41767-0

**Published:** 2019-03-29

**Authors:** Sven Neudeck, Andrey Mazilkin, Christian Reitz, Pascal Hartmann, Jürgen Janek, Torsten Brezesinski

**Affiliations:** 1 Battery and Electrochemistry Laboratory, Institute of Nanotechnology, Karlsruhe Institute of Technology (KIT), Hermann-von-Helmholtz-Platz 1, 76344 Eggenstein-Leopoldshafen, Germany; 2 Institute of Nanotechnology, Karlsruhe Institute of Technology (KIT), Hermann-von-Helmholtz-Platz 1, 76344 Eggenstein-Leopoldshafen, Germany; 3 Karlsruhe Nano Micro Facility, Karlsruhe Institute of Technology (KIT), Hermann-von-Helmholtz-Platz 1, 76344 Eggenstein-Leopoldshafen, Germany; 4 Institute of Solid State Physics, Russian Academy of Sciences, Ac. Ossipyan str. 2, 142432 Chernogolovka, Russia; 5 BASF SE, Carl-Bosch-Straße 38, 67056 Ludwigshafen, Germany; 6 Institute of Physical Chemistry & Center for Materials Science, Justus-Liebig-University Giessen, Heinrich-Buff-Ring 17, 35392 Giessen, Germany

## Abstract

Conformal coating of nm-thick Al_2_O_3_ layers on electrode material is an effective strategy for improving the longevity of rechargeable batteries. However, solid understanding of how and why surface coatings work the way they do has yet to be established. In this article, we report on low-temperature atomic layer deposition (ALD) of Al_2_O_3_ on practical, ready-to-use composite cathodes of NCM622 (60% Ni), a technologically important material for lithium-ion battery applications. Capacity retention and performance of Al_2_O_3_-coated cathodes (≤10 ALD growth cycles) are significantly improved over uncoated NCM622 reference cathodes, even under moderate cycling conditions. Notably, the Al_2_O_3_ surface shell is preserved after cycling in full-cell configuration for 1400 cycles as revealed by advanced electron microscopy and elemental mapping. While there are no significant differences in terms of bulk lattice structure and transition-metal leaching among the coated and uncoated NCM622 materials, the surface of the latter is found to be corroded to a much greater extent. In particular, detachment of active material from the secondary particles and side reactions with the electrolyte appear to lower the electrochemical activity, thereby leading to accelerated capacity degradation.

## Introduction

Ni-rich layered lithium transition-metal oxides, in particular LiNi_0.80_Co_0.15_Al_0.05_O_2_ (NCA) and Li_1+*x*_(Ni_1–*y*–*z*_Co_*y*_Mn_*z*_)_1–*x*_O_2_ (NCM, ≥60% Ni), are emerging as cathode active materials (CAMs) of choice for application in rechargeable lithium-ion batteries (LIBs) for electric vehicles^1,2^. Further optimization of these materials involves improving their mechanical and structural stability and suppressing side reactions such as corrosion. To this end, both bulk doping and coating the surface of secondary particles by a thin shell have been shown to be effective strategies^3–5^. Among the various types of coating materials that have been applied to CAMs, aluminum oxide (Al_2_O_3_) is by far the most prominent and widely used, not only in academia but also industry.

In general, Al_2_O_3_ can be prepared by solution (wet) chemistry^6–10^ or, as shown in recent years, via atomic layer deposition (ALD)^11–14^. Al_2_O_3_ ALD is a well-established process, usually consisting of two half-reaction steps using trimethylaluminum (TMA) and H_2_O, respectively, as the reactants. Major advantages of ALD over other techniques are the ability to produce conformal coating layers and the high degree of control over their thickness^15–17^. In most cases, ALD is applied to CAM powder as shown, for example, for LiCoO_2_ (LCO)^18–22^ and NCM^23–27^. However, ALD also offers the possibility to perform the coating directly on ready-to-use electrodes as reported for LCO^19,21,22,28–33^ and NCM523 (50% Ni)^34,35^, among others. In so doing, all electrode constituents (CAM, carbon black additive, polymer binder etc.) are covered by a coating and, thus, the entire accessible surface is protected from exposure to the electrolyte solution^36^. Because the contact points between the CAM secondary particles, the conductive carbon black and the current collector remain free of ALD material, superior electron and lithium-ion transport properties can be expected for coated electrodes over powder^19,22^. In addition, different processes and deposition conditions can be readily tested without time- and material-consuming preparation steps in between.

Despite the wide use of Al_2_O_3_ coatings in LIBs, a solid understanding of how and why they enhance the cycling performance has not yet been established. Several studies reported on reduced transition-metal dissolution after cycling and/or in storage^18,20,22,36^. However, Jung *et al*. have shown that, for NCM523, transition-metal dissolution is not the main source of degradation, even at high cell voltages (up to 4.8 V vs. Li^+^/Li)^37^. In agreement with the hypothesis of reduced transition-metal dissolution, lower HF concentrations in the electrolyte were observed for cells using Al_2_O_3_-coated cathodes^6,18^. This indicates that aluminum oxide-based surface shells undergo reaction with detrimental HF. In fact, time-of-flight secondary ion mass spectrometry revealed the formation of AlF_3_ and the corresponding AlO_*x*_F_*y*_ intermediate(s), which has been described as a kind of “HF (or H_2_O) scavenging” in the literature^6,18,38^. Nevertheless, studies on NCM523/Li cells cycled in the voltage range between 3.0 and 4.5 V reported the presence of Al_2_O_3_ after 100 cycles^34^, with no indication of the formation of fluorine species after 40 cycles^8^. Regardless of composition, an Al-containing layer was found on the CAM surface after cycling^24,38^, which, in some cases, appeared to be thinner than before^34,36^.

Because of the transition-metal dissolution, changes in CAM surface composition and morphology are apparently inevitable. However, Al_2_O_3_ coatings were reported to reduce or even prevent corrosion^6,24,38^. Furthermore, some studies concluded that irreversible transformations from layered to spinel^24,25^ and/or rock-salt-like phases^9,34^ at the CAM surface are inhibited by the presence of Al_2_O_3_. According to Jung *et al*. and Li *et al*., the formation of a detrimental surface layer in pristine NCM523 and NCM811, respectively, depends upon the upper cutoff voltage during cycling operation^37,39^.

So far, only few studies reported on the coating of technologically important CAMs such as Ni-rich NCM^24^. Besides, half-cells with a lithium-metal anode are usually tested and often only for a limited number of cycles at relatively harsh conditions (i.e., high temperature and/or cutoff voltage). The processes and phenomena observed in such experiments are not necessarily reflecting those prevailing in real-world applications. Overall, there is clearly a lack of long-term performance studies of graphite-based full-cells using ALD-modified CAM under reasonable cycling conditions.

Here, we applied the strategy of Al_2_O_3_ ALD to practical NCM622 (60% Ni) cathodes and examined their performance in LIB full-cells over hundreds of cycles. The leaching of transition-metal species from the CAM or, more rigorously, their deposition at the graphite anode and changes in bulk and surface structure were investigated by a combination of *operando* X-ray diffraction (XRD), inductively coupled plasma-optical emission spectroscopy (ICP-OES) and advanced electron microscopy.

## Results

### Electrode Preparation and Characterization

NCM622 composite cathodes were coated with Al_2_O_3_ using 4, 10 and 40 ALD growth cycles (referred to as ALD-4@NCM622, ALD-10@NCM622 and ALD-40@NCM622, respectively, hereafter). The deposition temperature was set to 110 °C to avoid thermal damage to the electrodes. The final ALD precursor exposure was always TMA. The reason is the detrimental effect of residual H_2_O and surface OH-groups on the cycling performance of LIB cells^40^. According to established growth-per-cycle data (~0.13 nm between 100 and 125 °C on Si substrate)^41^, the Al_2_O_3_ layer thickness was estimated to about 0.5, 1.3 and 5.3 nm for ALD-4@NCM622, ALD-10@NCM622 and ALD-40@NCM622, respectively.

ALD-40@NCM622 was used as a model material to ensure the ALD was successful and, more importantly, to determine whether the coating was homogeneous throughout the bulk of the cathode. Cross-sectional focused ion beam (FIB)-scanning electron microscopy (SEM) imaging and energy-dispersive X-ray spectroscopy (EDX) provided clear evidence of the presence of Al in the interior of electrodes (Supplementary Fig. 1). From this data, it appears that the material was primarily deposited on the surface of CAM secondary particles. Given that the total Al content is very low, even for ALD-40@NCM622, proper characterization of the Al_2_O_3_ coating required much higher spatial resolution. Thus, transmission electron microscopy (TEM) was conducted on the ALD-10@NCM622, as the surface shell of ALD-40@NCM622 is too thick for any practical use in LIBs.

**Figure 1:**
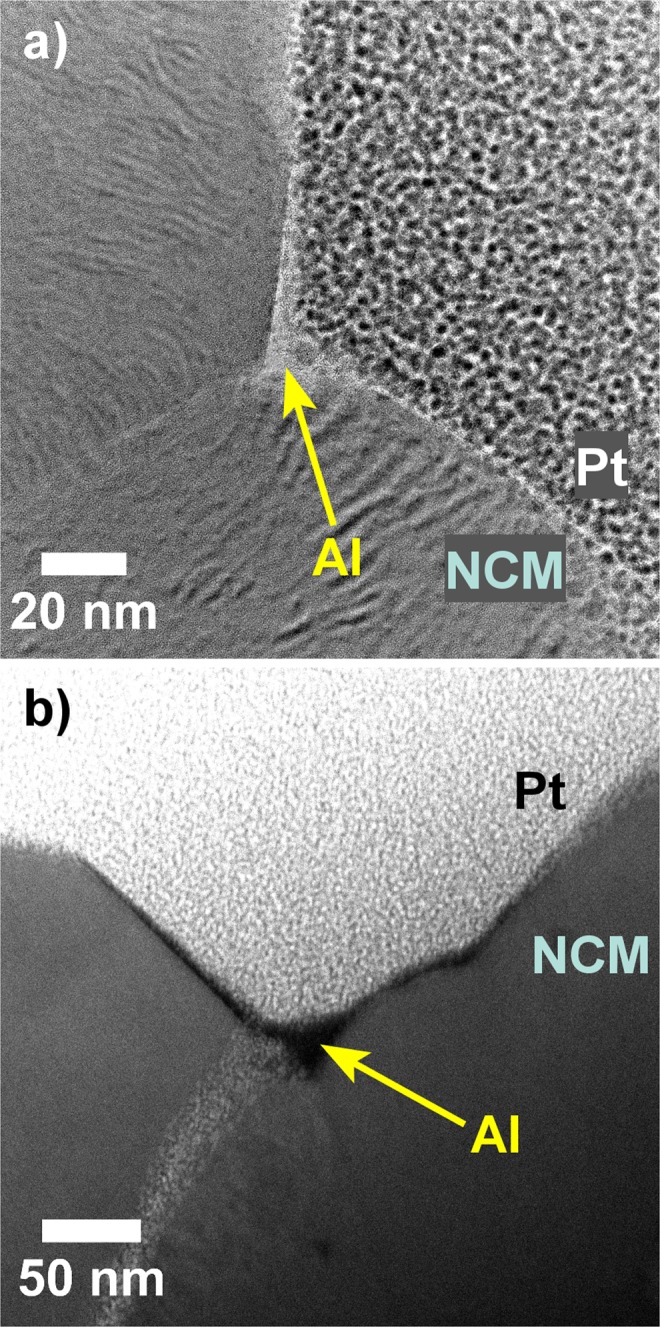
(**a**) BF-TEM and (**b**) HAADF-STEM images of ALD-10@NCM622. The electrode used was covered by a platinum (Pt) layer to protect the Al_2_O_3_ coating from damage during sample preparation and processing.

Both bright-field (BF)-TEM and *Z*-sensitive high-angle annular dark-field (HAADF)-scanning TEM (STEM) revealed the presence of a thin, amorphous layer on the top surface of secondary particles (Fig. 1 and Supplementary Fig. 2). The NCM622 CAM was rather uniformly covered with the layer thickness varying from 1 to 4 nm on flat parts of the surface. However, in areas where the surface is uneven, and especially at the contact points of particles, the coating was much thicker, even reaching a thickness of >10 nm. Such deviations from the expected value based on reported growth-per-cycle data were also observed for other substrates with “non-ideal” surfaces^21,42,43^, and explained by additional hydrogen-bonded H_2_O on the hydroxylated Al_2_O_3_, leading to a faster growth rate. Furthermore, it should be noted that the electrode itself may contain residual H_2_O from the preparation process, which readily reacts with the TMA precursor.

**Figure 2:**
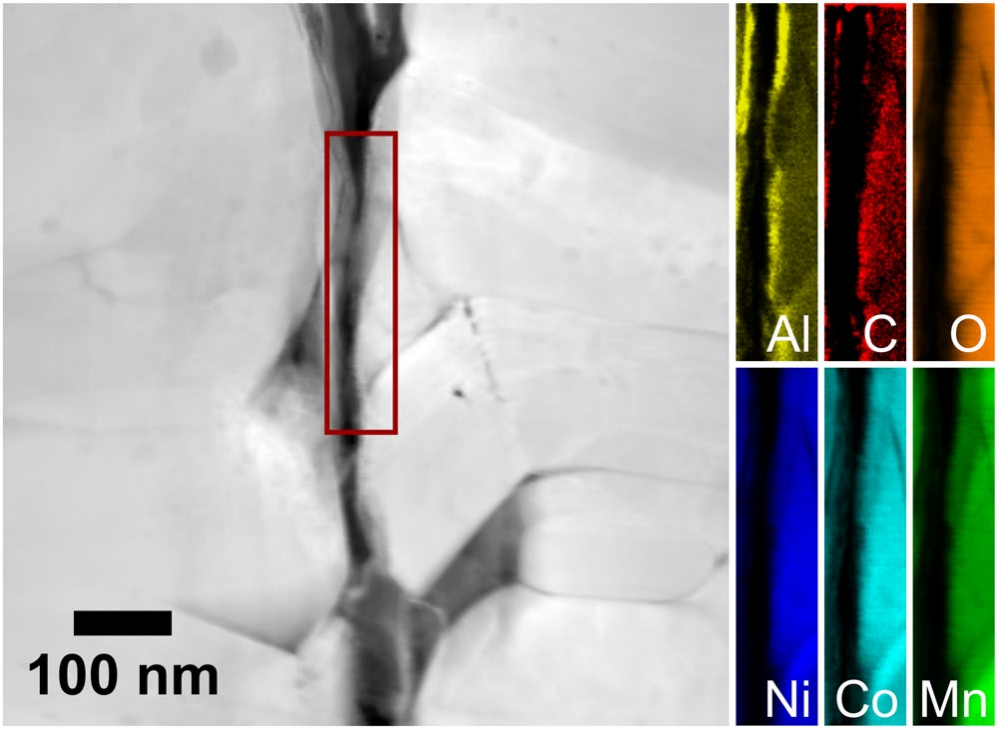
HAADF-STEM image of ALD-10@NCM622 and the corresponding EDX maps for the area between two adjacent CAM secondary particles denoted by the red box.

TEM-EDX mapping also corroborated the presence of an Al-based surface layer in the case of ALD-10@NCM622 (Fig. 2 and Supplementary Fig. 3). Because the Pt-M and Al-K lines are very close together, the default procedure for map extraction produced strongly overlapping signals. To distinguish between the two elements, the Al map was extracted using a narrow energy window at the signal position in the EDX spectrum.

### Cycling Performance

The electrochemical performance of the NCM622 CAM before and after Al_2_O_3_ ALD was evaluated at 25 °C in coin cells with both lithium-metal and graphite anodes. The initial specific discharge capacity at 0.1C of ALD-10@NCM622 half-cells [(166.7 ± 0.4) mAh/g_NCM_] was found to be slightly lower than that of both ALD-4@NCM622 [(167.5 ± 0.2) mAh/g_NCM_] and bare NCM622 [(167.9 ± 0.3) mAh/g_NCM_]. This result indicates that about 1 mAh/g_NCM_ of discharge capacity is lost due to surface coating when using 10 ALD growth cycles. Part of the reason is the lower active material content.

The mass of ALD-derived Al_2_O_3_ can be estimated assuming that the difference in discharge capacity is only due to differences in CAM content. When further considering the specific surface area of the bare NCM622 from gas adsorption (0.47 m^2^/g_NCM_) and the solid-state density of Al_2_O_3_ (3.97 g/cm^3^)^44^, the average shell thickness can be calculated as 4 nm for ALD-10@NCM622, which agrees with the TEM results. Besides, it should be noted that the difference in discharge capacity can probably also be attributed to lithiation of the Al_2_O_3_ layer^45,46^.

The rate capability was tested by charging the half-cells at 0.25C and discharging them at rates ranging from 0.1 to 3C (Supplementary Fig. 4). Hardly any differences in specific discharge capacity within the experimental error were observed between ALD-4@NCM622 and bare NCM622, whereas the cells using ALD-10@NCM622 delivered overall lower capacities, especially at rates ≥1C, thus indicating that the thicker Al_2_O_3_ coating somewhat hinders charge transfer. Furthermore, the data demonstrate that the ALD-coated NCM622 CAMs are capable of outperforming the reference material in terms of capacity retention. After 100 cycles, both the ALD-10@NCM622 and ALD-4@NCM622 cells delivered discharge capacities higher by about 2 mAh/g_NCM_ than the bare NCM622. In summary, we conclude that, regardless of thickness, Al_2_O_3_ coating on NCM622 composite cathodes leads to improved capacity retention. However, a relatively thicker surface shell results in lower initial discharge capacity and rate capability. Thus, in terms of absolute capacity and kinetics, the ALD layer should be as thin as possible.

**Figure 3:**
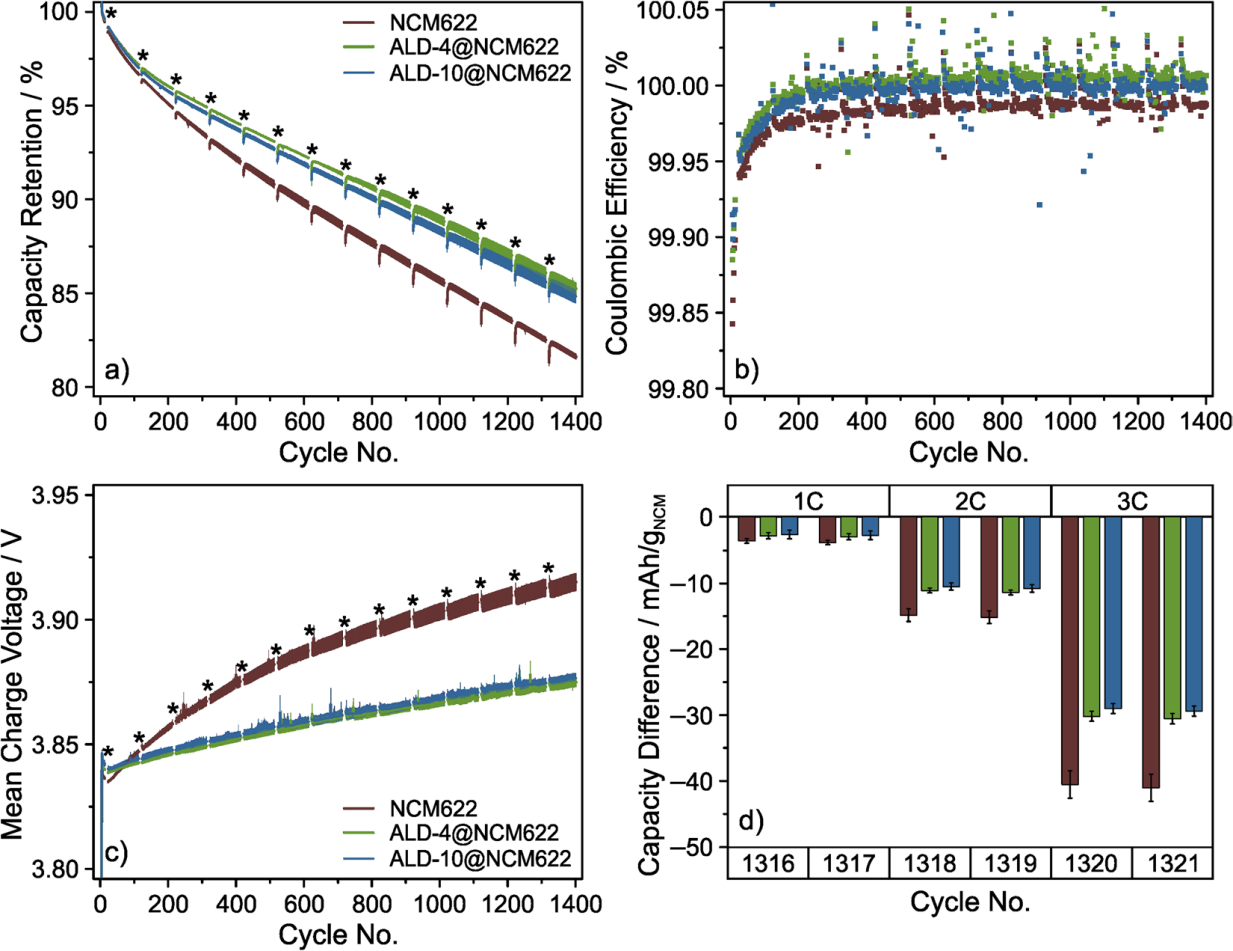
(**a**) Capacity retention relative to 5^th^ cycle discharge capacity at 1C rate, (**b**) Coulombic efficiency and (**c**) mean charge voltage versus cycle number as well as (**d**) rate capability in the later cycles of graphite-based full-cells using bare NCM622, ALD-4@NCM622 and ALD-10@NCM622. After the initial formation cycles at 0.1C were completed, the cells were charged and discharged at 1C, with a rate performance test every 100 cycles (denoted by asterisks in (**a**,**c**); data omitted for clarity), during which they were charged at 0.5C and discharged at rates up to 3C. Differences in specific discharge capacity relative to that at 0.5C are shown in (**d**). Cycling results are averaged from several cells, with the error bars indicating the standard deviation of the mean.

The graphite-based full-cells were cycled at a rate of 1C in the voltage range between 2.8 and 4.2 V with a constant voltage (CV) step at the upper cutoff voltage. A rate capability test was implemented every 100 cycles. Figure 3 and Supplementary Fig. 5 depict cycling data averaged from at least 4 independent experiments (cells), thereby allowing for fair comparison, especially of the ALD-coated NCM622 CAMs. As expected, all cells delivered similar initial discharge capacities of (164 ± 1) mAh/g_NCM_ at 0.1C. At 1C rate, the discharge capacity of both the ALD-4@NCM622 and ALD-10@NCM622 cells was found to be slightly lower for the first 200 to 300 cycles (Supplementary Fig. 5). The capacity decay in the subsequent cycles was almost linear for the different CAMs employed in this work. As for capacity retention, ALD-4@NCM622 and ALD-10@NCM622 clearly outperformed the bare NCM622 (Fig. 3a). After 1400 cycles, the ALD-10@NCM622 cells still delivered a specific discharge capacity of (127.2 ± 0.6) mAh/g_NCM_, corresponding to (84.8 ± 0.4)% relative to the 5^th^ cycle capacity at 1C. While similar results were obtained for ALD-4@NCM622 [(126.5 ± 0.4) mAh/g_NCM_, (85.3 ± 0.4)%], the performance of the bare NCM622 reference was considerably worse [(123.3 ± 0.2) mAh/g_NCM_, (81.6 ± 0.2)%].

To better illustrate the improvement in longevity achieved by Al_2_O_3_ ALD coating, the cycle numbers, at which the cell capacity faded to 85% of its rated value, were compared. In fact, this was found to occur much earlier for bare NCM622, after 1069 cycles, compared to 1381 cycles for ALD-10@NCM622. Considering the capacity decay rates, the difference in cycle number between the cells is projected to be even larger for 80% capacity retention, which is typically defined as end-of-life of commercial battery cells^47^.

The Coulombic efficiency of all cells increased to > 99.95% within the first 20 cycles (Fig. 3b). As anticipated based on the above data, the Coulombic efficiency of the bare NCM622 cells was slightly lower than that of cells using the ALD-coated CAMs over the entire 1400 cycles. The cell impedance reflected the same trend. During the first 70 cycles, both the ALD-4@NCM622 and ALD-10@NCM622 cells exhibited a higher resistance, expressed by higher mean charge voltages (Fig. 3c). This is clearly due to the presence of Al_2_O_3_ coating on the composite cathodes, which apparently raises the energy barrier for (ionic) charge transfer. However, a much stronger increase in mean charge voltage with prolonged cycling was observed for the bare NCM622. Again, there was no significant difference between the ALD-4@NCM622 and ALD-10@NCM622 cells. Other measures of cell resistance such as mean discharge voltage and specific capacity gained in the CV step during charging revealed similar trends (Supplementary Fig. 5).

The effect of Al_2_O_3_ ALD on the kinetics was also somewhat reflected in the capacity retention data (see temporary capacity decline right after rate performance testing during cycling). Furthermore, the rate capability results agreed with the trends seen in cycling performance and impedance buildup (Fig. 3d and Supplementary Fig. 6). During the first test after around 10 cycles, the extent of capacity decrease at rates ≥2C was found to be slightly lower for bare NCM622 than the ALD-coated CAMs. At 3C, the specific discharge capacity of the bare NCM622 cells was lower by (20.5 ± 0.4) mAh/g_NCM_ relative to that delivered at 0.5C rate ([23.0 ± 0.4] mAh/g_NCM_ for ALD-4@NCM622 and [22.6 ± 0.5] mAh/g_NCM_ for ALD-10@NCM622). However, this trend was reversed after 200 cycles (e.g., [41 ± 2] mAh/g_NCM_ for bare NCM622, compared to [30.5 ± 0.8] mAh/g_NCM_ for ALD-4@NCM622 and [29.3 ± 0.8] mAh/g_NCM_ for ALD-10@NCM622 at 3C rate after around 1300 cycles; see Fig. 3d).

The data discussed thus far clearly establish that Al_2_O_3_ ALD coating of NCM622 composite cathodes brings about significant improvements in stability and cycling performance of LIB full-cells. Initially higher resistances and slower kinetics are quickly compensated for by a much weaker tendency for impedance buildup compared to the bare NCM622 cells. Note that the number of ALD growth cycles (when ≥ 4 and ≤ 10) apparently does not play a major role in controlling the cyclability.

### Analysis of Failure Modes

To understand how ALD coating contributes to the performance improvement, electrodes harvested from cells after cycling for 1400 cycles were investigated by ICP-OES, *operando* XRD and TEM. Transition-metal leaching from the CAM particles is often claimed to be responsible (to some extent) for the capacity decay^48,49^. In addition to loss of CAM, especially loss of active lithium, which is consumed during rebuilding of the graphite solid electrolyte interphase (SEI) due to continuous destruction/poisoning, needs to be considered^50,51^. The amount of transition-metal species deposited at the anode side was determined via ICP-OES (Supplementary Fig. 7). Interestingly, the proportion of Mn was similar to that of Ni (about 0.6 mg/g_graphite_), which is certainly due in part to the higher dissolution rate of Mn than both Co and Ni. However, the total amount is too small to explain the observed capacity losses. Most importantly, there were no significant differences between the cells. Consequently, transition-metal dissolution and subsequent deposition are unlikely to be the main reason for the different capacity fade rates. This result is in agreement with data reported for NCM523^37^. It should be noted though that the type of separator used can greatly affect the transition-metal deposition on the anode (e.g., glass fiber separators can potentially reduce the transition-metal leaching by reacting with HF present in the electrolyte). Likewise, *operando* XRD did not indicate major differences with respect to bulk composition and crystallinity between the ALD-10@NCM622 and bare NCM622. The changes in *a* and *c* lattice parameters and, thus, also of unit cell volume upon Li insertion/extraction into/from the NCM lattice follow the trends observed in recent studies on Ni-rich NCMs and are more or less identical for both CAMs (Supplementary Fig. 8)^52–57^.

**Figure 4:**
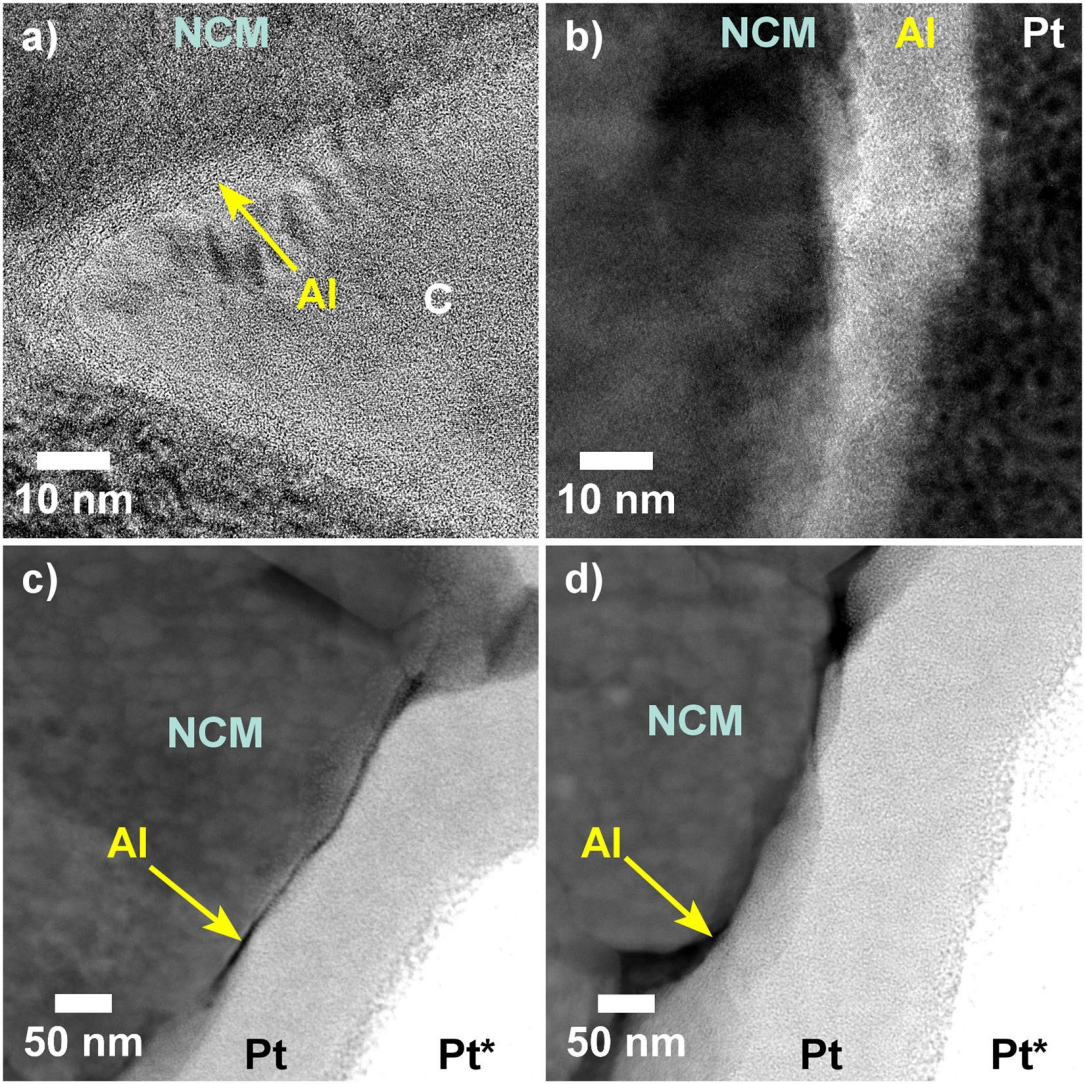
(**a**,**b**) HR-TEM and (**c**,**d**) HAADF-STEM images of ALD-10@NCM622 after cycling in full-cell configuration for 1400 cycles. The electrodes used were covered by carbon (C) or platinum protective layers, the latter of which were produced by electron-beam (Pt) and ion-beam (Pt*) assisted deposition.

Finally, the electrodes were characterized using TEM. Notably, even after cycling in full-cell configuration for 1400 cycles, an Al-containing surface layer was present on the ALD-10@NCM622 as evidenced by high-resolution (HR-)TEM and HAADF-STEM (Fig. 4). This result was further confirmed by TEM-EDX mapping (Supplementary Fig. 9). Although Al_2_O_3_ coating is widely used in the LIB area, only a limited number of studies reported experimental data on the robustness of the surface shell^6,24,34,38^. Unfortunately, Al_2_O_3_ and AlF_3_, the latter of which is believed to form by reaction with HF during cycling operation^6,18,38^, could not be distinguished by EDX analysis. The reason is that the F-K line and the Co-L and Mn-L series are close together and background subtraction in this energy range is difficult. Thus, it remains unclear whether or not Al_2_O_3_ acted as an “HF scavenger” in the present work.

**Figure 5:**
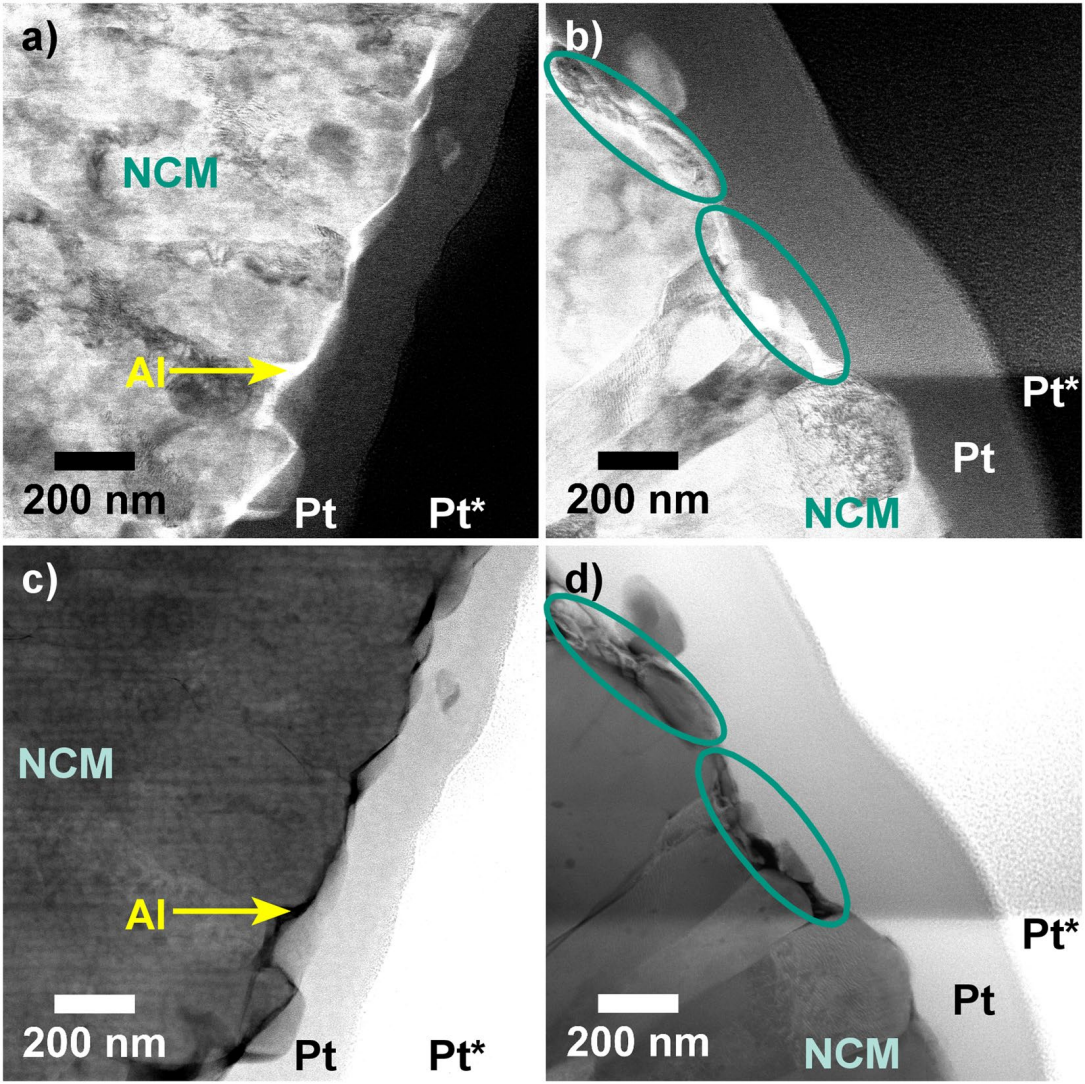
(**a**,**b**) BF-STEM and (**c**,**d**) HAADF-STEM images of ALD-10@NCM622 (**a**,**c**) and bare NCM622 (**b**,**d**) after cycling in full-cell configuration for 1400 cycles. Areas of pronounced surface degradation are denoted by green ellipses. The electrodes used were covered by platinum protective layers, produced by electron-beam (Pt) and ion-beam (Pt*) assisted deposition.

**Figure 6:**
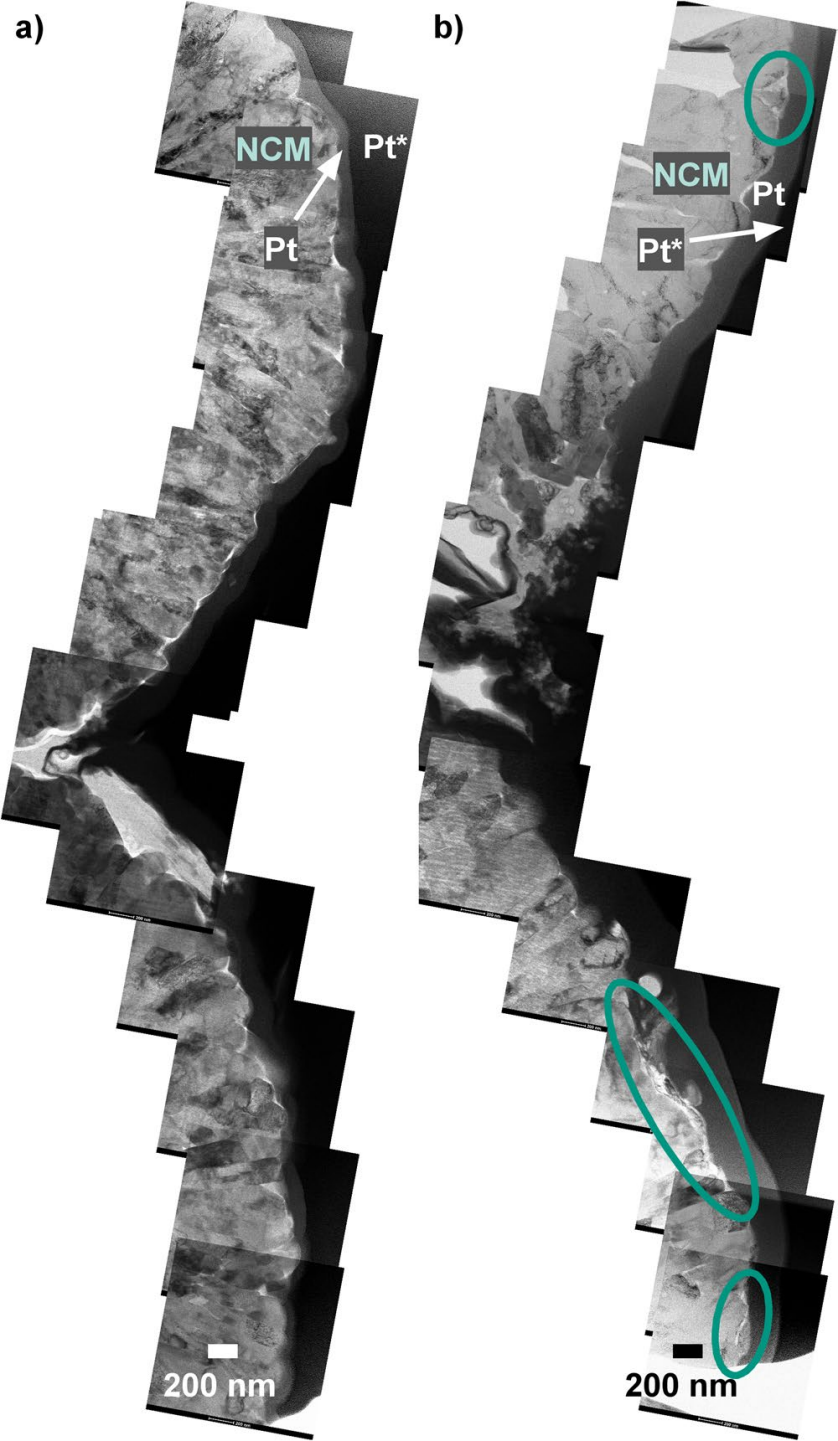
BF-STEM images of ALD-10@NCM622 (**a**) and bare NCM622 (**b**) after cycling in full-cell configuration for 1400 cycles. The top surface of two adjacent secondary particles is reconstructed by stringing together overlapping images. Areas of pronounced surface degradation are denoted by green ellipses. The electrodes used were covered by platinum protective layers, produced by electron-beam (Pt) and ion-beam (Pt*) assisted deposition. Corresponding HAADF-STEM images are shown in Supplementary Fig. 13.

Direct comparison of the ALD-coated and bare NCM622 CAMs cycled under identical conditions revealed distinct differences in surface morphology (Fig. 5). The reference material appeared to be corroded to a much greater extent. The ALD-10@NCM622 top surface was relatively smooth with uniform primary particles. In the case of bare NCM622, the presence of particles of much smaller size than the primary particles and without the typical radial orientation was observed. According to TEM-EDX mapping, these particles are made of transition-metal oxides (Supplementary Figs 10 and 11). However, as revealed by HAADF-STEM, lighter elements, probably arising from decomposition of the electrolyte solvents and the supporting salt, fill much of the intergranular space between them. In fact, both carbon and phosphorus were detected by EDX. Apparently, the NCM622 CAM is partially dissolved during the corrosion process and the empty space is filled with decomposition products from side reactions. Reprecipitation of solutes cannot be ruled out and might help explain the formation of smaller particles. This kind of corrosion was observed in many areas on the surface of bare NCM622 secondary particles as indicated by BF-STEM and HAADF-STEM measurements (Fig. 6 and Supplementary Figs 12 and 13). Both detachment of CAM and probably also of carbon black additive and polymer binder from the secondary particles and formation of a cathode SEI lead to loss of electrochemical activity and, therefore, capacity degradation.

Lastly, we note that close inspection of fast Fourier transform patterns of HR-TEM images did not indicate the presence of rock-salt-like phases. In addition, electron energy-loss spectroscopy core-loss spectra of transition-metal-L edges obtained on the ALD-10@NCM622 at different areas of the primary particles did not reveal signs of chemical shifts, which may be expected for a layered-to-rock-salt transformation^58,59^. However, as mentioned previously^37,39^, the formation of rock-salt structure on the surface of Ni-rich NCM CAMs apparently depends on the cutoff voltage in the charge cycle and may be inhibited or even prevented by the presence of Al_2_O_3_ surface shells. Nevertheless, this needs further study.

We emphasize once again that the general cycling conditions (separator type, volume of electrolyte in the cell, cutoff voltage etc.) have a profound effect on the degradation processes occurring in LIB cells. For example, the glass fiber separator used here can potentially react with the HF in the electrolyte, thus indirectly affecting the cell chemistry. By rigorously comparing different materials under identical conditions, as done in this study, we identified the surface corrosion as being a major cause of degradation in the case of uncoated NCM622. Other mechanisms, triggered by particle fracture, transition-metal deposition on the anode or formation of rock-salt-like phases on the CAM particle surface, to name a few, may come into play under different conditions. Thus, further work is clearly necessary to fully understand the specific degradation pathways and the role of thin surface coatings in the battery area.

## Discussion

NCM622 composite cathodes of practical loading were successfully coated by Al_2_O_3_ ALD. The coating thickness was controlled by varying the number of ALD growth cycles. Characterization of the modified cathodes via electron microscopy and X-ray spectroscopy revealed that Al_2_O_3_, which appears to be primarily deposited on the surface of secondary particles, is present throughout the bulk of the electrode.

Electrochemical testing was performed in half- and full-cells. The Al_2_O_3_-coated NCM622 clearly outperformed the uncoated reference material in terms of capacity retention and efficiency (e.g., much lower cell impedance growth with cycling), even under moderate conditions. In graphite-based cells, the number of ALD growth cycles when ≥4 and ≤10 was found to not play a major role in controlling the performance and rate capability. Transmission electron microscopy studies comparing the coated and uncoated NCM622 demonstrated that the Al_2_O_3_ surface shell is well preserved after cycling in full-cell configuration for 1400 cycles and, even more importantly, effectively reduces corrosion. Only the top surface of ALD-coated NCM622 appeared smooth and largely intact, correlating with the observed cycling performance and helping to explain the improved longevity. There were hardly any differences in bulk lattice structure and transition-metal leaching between the coated and uncoated NCM622. Furthermore, no clear evidence of surface phase transformations for a charging voltage of 4.2 V vs. graphite was found. Taken together, our data establish that the cathode-electrolyte interphase stability is greatly enhanced by Al_2_O_3_ ALD.

## Methods

### Electrode Preparation

Solef5130 polyvinylidene fluoride (PVDF) binder was dissolved in *N*-methyl-2-pyrrolidone (NMP, Merck; Germany) to prepare a 7.5 wt.% solution. For the preparation of electrodes, binder (3 wt.%) solution, SFG6L graphite (2 wt.%) and Super C65 carbon black (1 wt.%) were suspended in NMP. After mixing for 3 min at 2000 rpm and then for another 3 min at 400 rpm using an ARE 250 planetary centrifugal mixer (Thinky Corp.; Japan), NCM622 CAM (94 wt.%, BASF SE; Germany) was added. The suspension was mixed twice at the above conditions to obtain the final slurry. The solid content was adjusted to 65% by addition of extra NMP during mixing in the first place. The slurry was coated onto aluminum foil using a doctor blade on a film applicator (Coatmaster 510, Erichsen GmbH & Co. KG; Germany). After drying in vacuum at 120 °C (VDL 53, Binder GmbH; Germany), the cathodes were calendared at 10 N/mm using a laboratory calendar (Sumet Systems GmbH; Germany). The areal loading was 11.6–11.7 mg_NCM_/cm^2^.

### ALD Coating

Al_2_O_3_ ALD on NCM622 composite cathodes was performed on a SUNALE R-200 Advanced (Picosun; Finland). The electrodes were mounted on an 8″ Si(001) wafer and then heated to 110 °C. The two half-reaction steps consisted of injecting five times TMA or H_2_O with 0.1 s purging between each pulse and purging of 40 s after the last pulse. 4, 10 and 40 ALD growth cycles were used to prepare the Al_2_O_3_-coated cathodes.

### Cell Assembly

CR2032 coin cells (Hohsen Corp.; Japan) were assembled inside an argon-filled glovebox (MBraun; Germany) using materials pre-dried at 100 °C in vacuum. For half-cells, NCM622 cathode (Ø14 mm), lithium-metal anode (Ø15 mm, Gelon LIB Co.; China) and glass fiber separator (GF/D Whatman, GE Healthcare Life Sciences; USA) soaked with electrolyte solution (200 µL LP47 [1.0 M LiPF_6_ in 3:7 by weight ethylene carbonate (EC)/diethyl carbonate (DEC)], BASF SE; Germany) were used.

Full-cells were assembled using NCM622 cathode (Ø14 mm), graphite anode (Ø15 mm, 6.8 mg_graphite_/cm^2^, BASF SE; Germany) and glass fiber separator (GF/A Whatman) soaked with electrolyte solution (100 µL LP472 [LP47 containing 2 wt.% vinylene carbonate], BASF SE; Germany). All cells were sealed using an MSK-160D crimper (MTI Corp.; USA).

### Cell Disassembly

Discharged cells were de-crimped inside the glovebox. The cathode was harvested and rinsed with DEC (4 × 250 µL, Sigma-Aldrich; Germany). After soaking in DEC (500 µL), the separator and graphite anode were carefully separated and the latter was washed with DEC (2 × 250 µL).

### Cell Testing

Electrochemical testing was performed at 25 °C on a Series 4000 multichannel battery cycler (MACCOR Inc.; USA). After equilibration for 2 h, half-cells were cycled between 3.0 and 4.3 V with a constant voltage (CV) step at the upper cutoff voltage, followed by a 5 min resting period prior to discharge. During the initial three cycles at 0.1C (1C = 160 mA/g_NCM_), the CV step was limited either by time (30 min) or by residual current (0.01C). The cells were then charged at 0.25C, with the CV step limited to 20 min or 0.05C, and discharged at different rates from 0.1 to 3C for three cycles each. Eventually, the discharge rate was set to 0.5C. Half-cell data are averaged from two (for ALD-4@NCM622) and three independent cells (for bare NCM622 and ALD-10@NCM622).

After equilibration for 6 h, full-cells were cycled between 2.8 and 4.2 V with a CV step at 4.2 V. The CV step was also limited by time (1 h) or residual current (0.02C), followed by a 5 min resting period. After three formation cycles at 0.1C were completed, the cells were cycled at 1C, with a rate performance test every 100 cycles (charging at 0.5C and discharging at different rates of 0.5, 1, 2 and 3C for two consecutive cycles). Long-term cycling data are averaged from four (for ALD-4@NCM622 and ALD-10@NCM622) and seven independent cells (for bare NCM622).

### Instrumentation

The content of transition-metal species in/on the graphite anode after cycling was determined by ICP-OES using both a PerkinElmer Optima 4300 DV and a Thermo Scientific iCAP 7600. ICP-OES data are averaged from three independent cells. FIB-SEM-EDX was performed on a ZEISS Auriga 60. TEM was performed on both FEI Tecnai F20 and TITAN 60–300 microscopes. To protect the Al_2_O_3_ coating from damage during sample preparation and later processing, the specimens were covered by a layer of carbon and/or platinum produced by initial electron-beam and subsequent ion-beam assisted deposition using an FEI Strata Dual Beam instrument. After formation of the protective layer(s), TEM specimens were prepared by gallium focused ion-beam milling. XRD was performed in transmission mode (300 s exposure time) on a custom Mo-K_α1,2_ diffractometer. The intensity of two consecutive 2D patterns was added up and integrated, yielding high-quality 1D data for analysis.

## Supplementary information

Supplementary Information

## Data Availability

All data generated or analyzed during this study are included in this published article (and its Supplementary Information files).
