# Supplementary material for: Effect of Low-Temperature Al2O3 ALD Coating on Ni-Rich Layered Oxide Composite Cathode on the Long-Term Cycling Performance of Lithium-Ion Batteries
Source: Sci Rep. 2019 Mar 29;9:5328. doi: 10.1038/s41598-019-41767-0 (PMC6441043; doi:10.1038/s41598-019-41767-0)
Supplement: Supplementary file 1 — Supplementary Information [file 41598_2019_41767_MOESM1_ESM.pdf]

Supplementary Information

Effect of Low-Temperature Al_2O_3 ALD Coating on Ni-Rich Layered Oxide Composite Cathode on the Long-Term Cycling Performance of Lithium-Ion Batteries

Sven Neudeck,*[1] Andrey Mazilkin,[1234] Christian Reitz,[23] Pascal Hartmann,[15] Jürgen Janek[16] & Torsten Brezesinski*[1]

[1]Battery and Electrochemistry Laboratory, [2]Institute of Nanotechnology, Karlsruhe Institute of Technology (KIT), Hermann-von-Helmholtz-Platz 1, 76344 Eggenstein-Leopoldshafen, Germany.

[3]Karlsruhe Nano Micro Facility, Karlsruhe Institute of Technology (KIT), Hermann-von-Helmholtz-Platz 1, 76344 Eggenstein-Leopoldshafen, Germany.

[4]Institute of Solid State Physics, Russian Academy of Sciences, Ac. Ossipyan str. 2, 142432 Chernogolovka, Russia.

[5]BASF SE, Carl-Bosch-Straße 38, 67056 Ludwigshafen, Germany.

[6]Institute of Physical Chemistry & Center for Materials Science, Justus-Liebig-University Giessen, Heinrich-Buff-Ring 17, 35392 Giessen, Germany.

Correspondence and requests for materials should be addressed to S.N. (sven.neudeck@kit.edu) or T.B. (torsten.brezesinski@kit.edu).

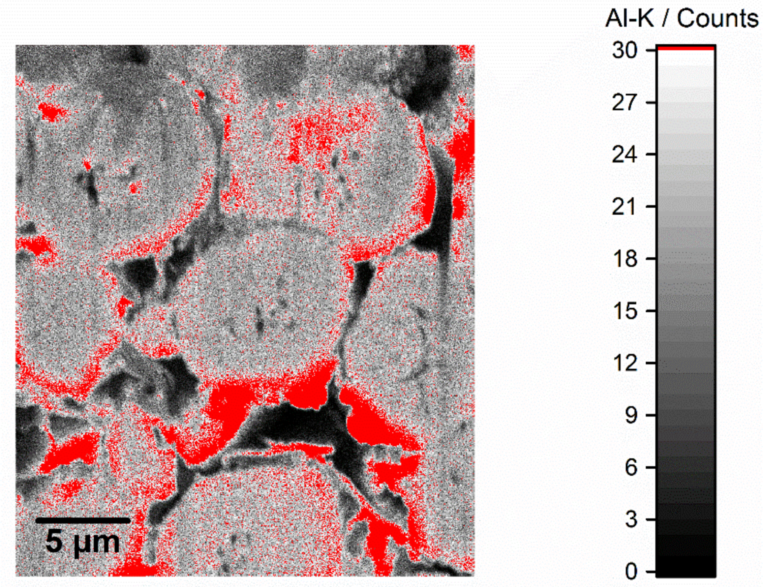

Figure S1. Cross-sectional FIB-SEM-EDX of ALD-40@NCM622. The electrode was not protected against damage that may occur during sample preparation.

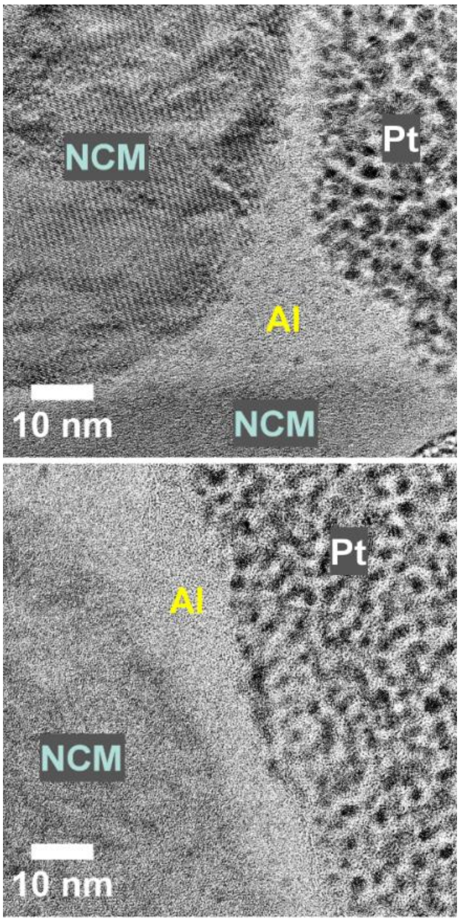

Figure S2. HR-TEM images of ALD-10@NCM622. The specimen was covered by a Pt layer to protect the Al_2O_3 coating from damage during sample preparation and processing.

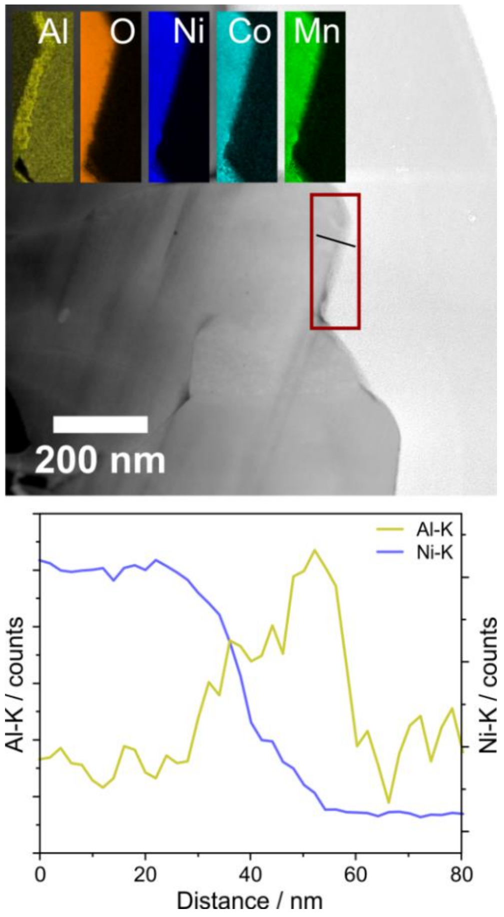

Figure S3. Top: HAADF-STEM image of ALD-10@NCM622 and the corresponding EDX maps for the area denoted by the red box. Bottom: EDX profiles for Al and Ni along the black line shown in the red box.

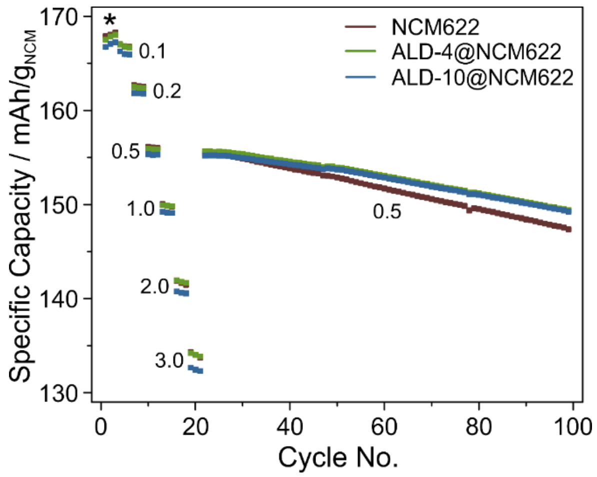

Figure S4. Cycling performance of half-cells using bare NCM622, ALD-4@NCM622 and ALD-10@NCM622. After three cycles at 0.1C were completed (denoted by an asterisk), the cells were charged at 0.25C and discharged at rates from 0.1 to 3C. Note that only specific discharge capacities averaged from at least two independent cells, with the error bars indicating the standard deviation of the mean, are shown.

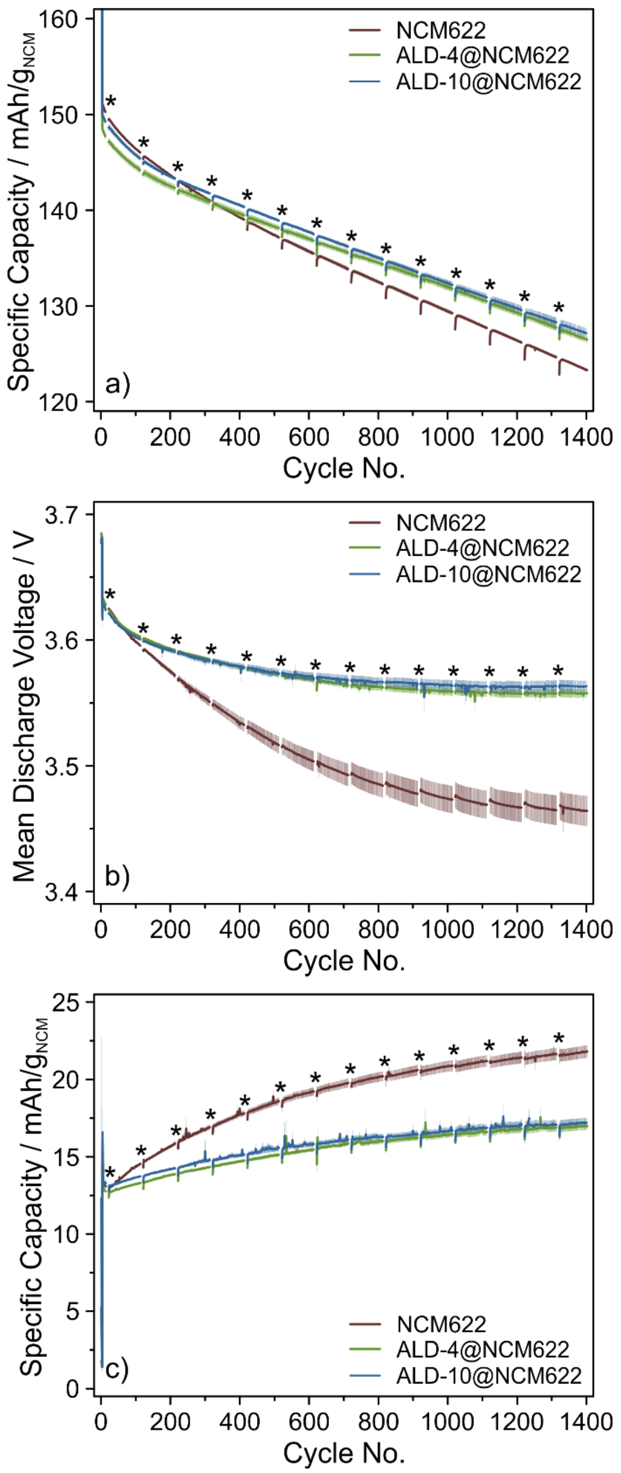

Figure S5. Cycling performance of graphite-based full-cells using bare NCM622, ALD-4@NCM622 and ALD-10@NCM622. (a) Specific discharge capacity, (b) mean discharge voltage and (c) specific capacity gained in the CV step at the upper cutoff voltage versus cycle number. After the initial formation cycles at 0.1C were completed, the cells were charged and discharged at 1C, with a rate capability test every 100 cycles (denoted by asterisks; data omitted for clarity). Note that the cycling data are averaged from several cells, with the error bars indicating the standard deviation of the mean.

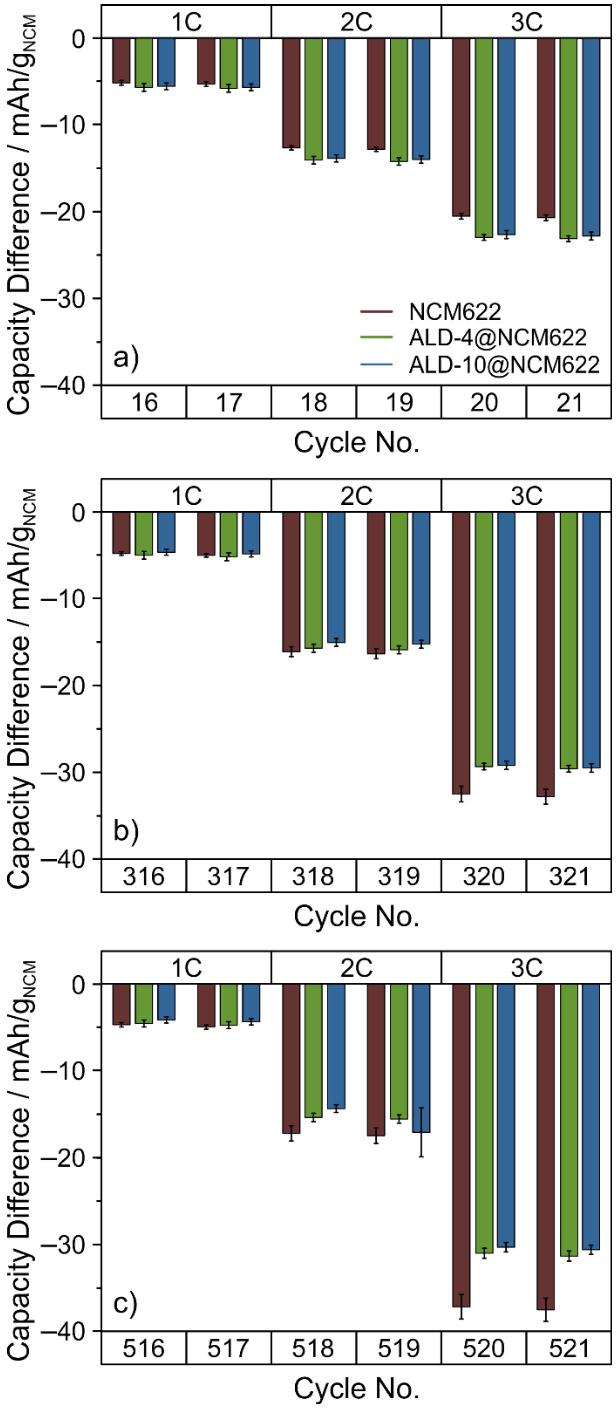

Figure S6. Rate performance testing (a) in early and (b,c) later cycles of graphite-based full-cells using bare NCM622, ALD-4@NCM622 and ALD-10@NCM622. The cells were charged at 0.5C and discharged at different rates of 0.5, 1, 2, and 3C for two cycles each. The differences in specific capacity relative to that at 0.5C, averaged from several cells with the error bars indicating the standard deviation of the mean, are shown.

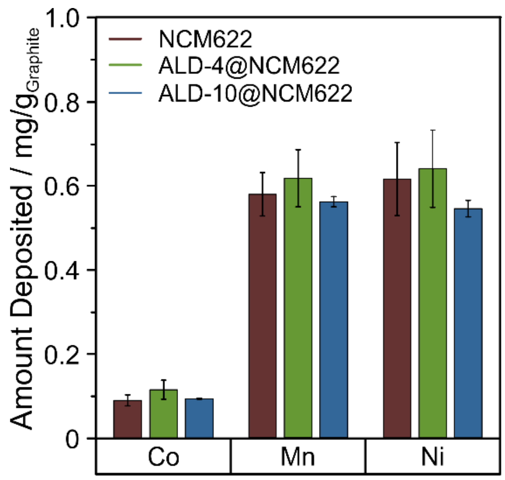

Figure S7. Transition-metal deposition at the anode side of graphite-based full-cells using bare NCM622, ALD-4@NCM622 and ALD-10@NCM622. Amounts of Co, Mn and Ni species from ICP-OES after cycling in full-cell configuration for 1400 cycles. Error bars indicate the standard deviation of the mean.

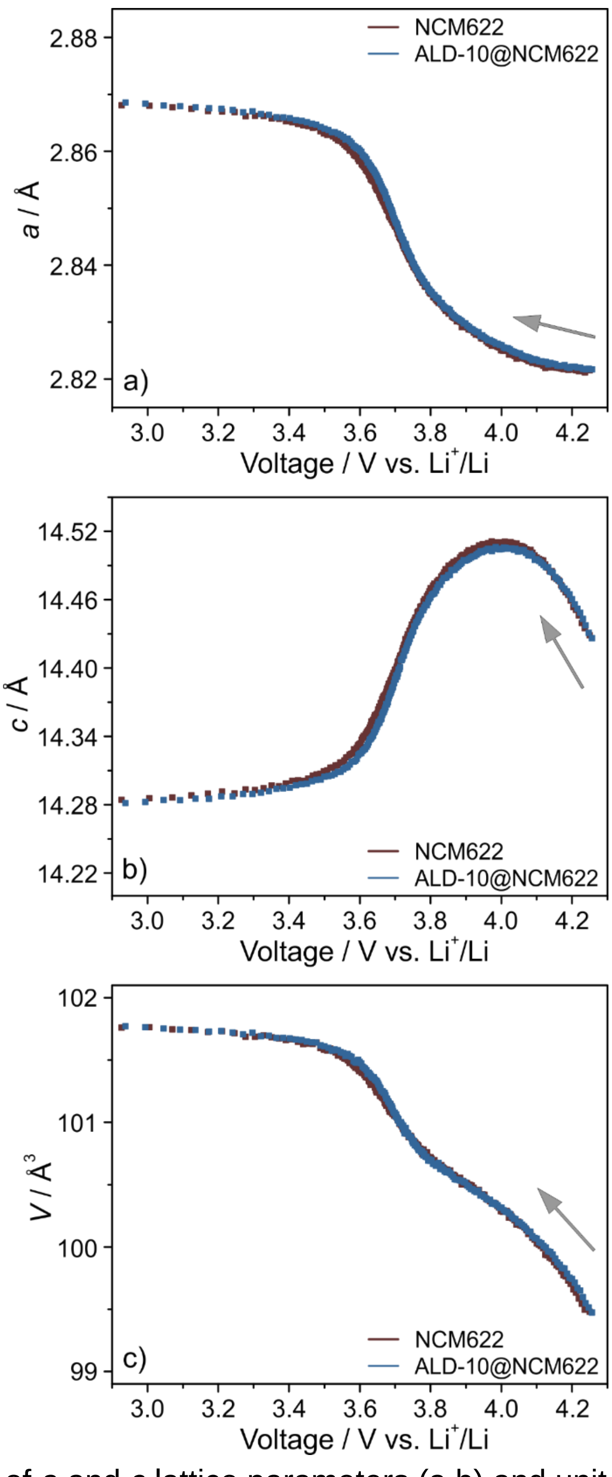

Figure S8. Evolution of *a* and *c* lattice parameters (a,b) and unit cell volume (c) of bare NCM622 and ALD-10@NCM622 after cycling in full-cell configuration for 1400 cycles. *Operando* XRD was performed on half-cells. Note that only the discharge cycle (lithiation of NCM) at C/10 rate is depicted.

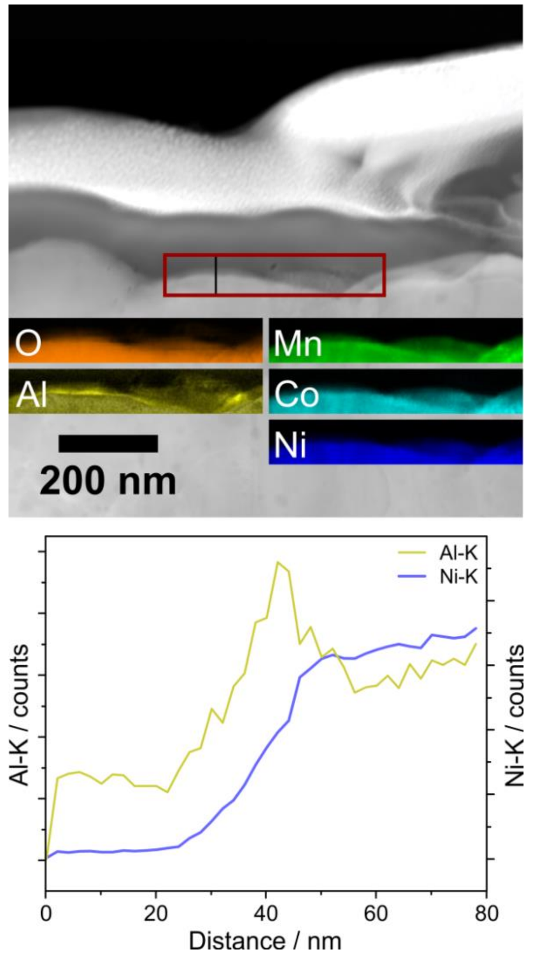

Figure S9. Top: HAADF-STEM image of ALD-10@NCM622 after cycling in full-cell configuration for 1400 cycles and the corresponding EDX maps for the area denoted by the red box. Bottom: EDX profiles for Al and Ni along the black line shown in the red box. The sample was covered by carbon/platinum protective layers.

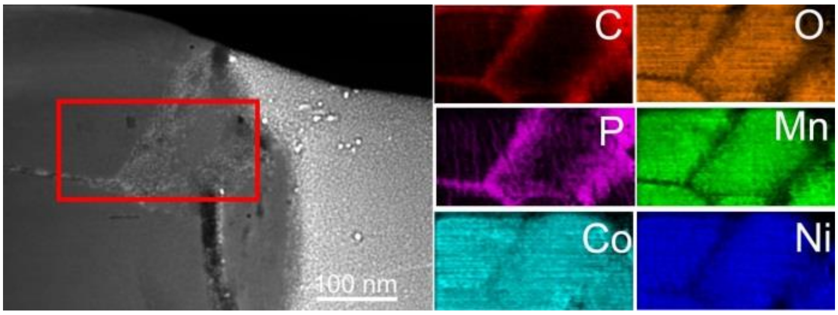

Figure S10. HAADF-STEM image of bare NCM622 after cycling in full-cell configuration for 1400 cycles and the corresponding EDX maps for the area denoted by the red box.

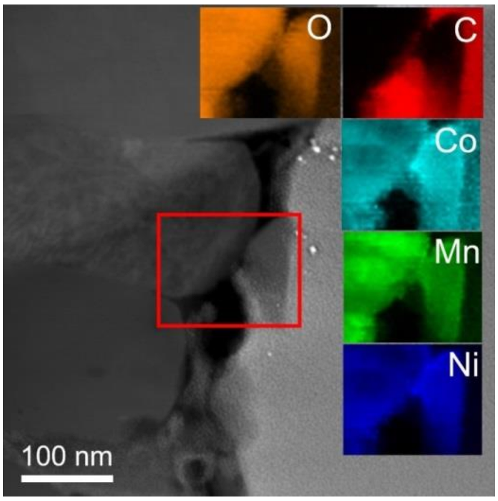

Figure S11. HAADF-STEM image of bare NCM622 after cycling in full-cell configuration for 1400 cycles and the corresponding EDX maps for the area denoted by the red box.

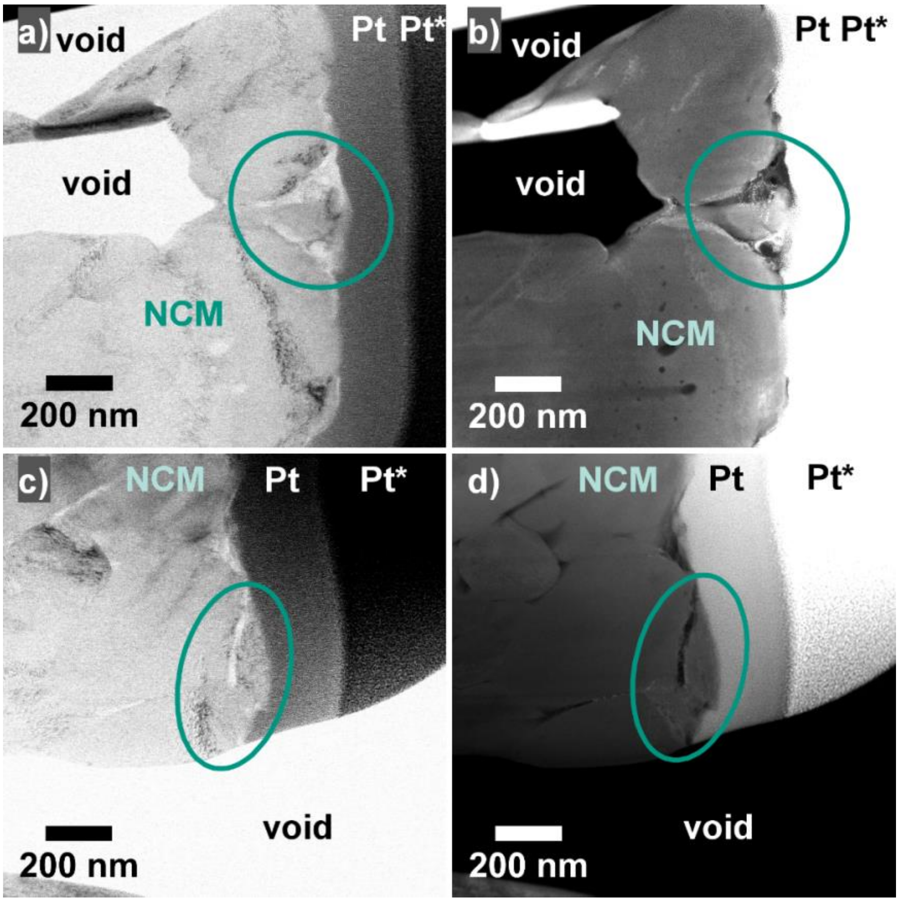

Figure S12. (a,c) BF-STEM and (b,d) HAADF-STEM images of bare NCM622 after cycling in full-cell configuration for 1400 cycles. Areas of pronounced surface degradation are denoted by green ellipses. The electrodes were covered by platinum protective layers.

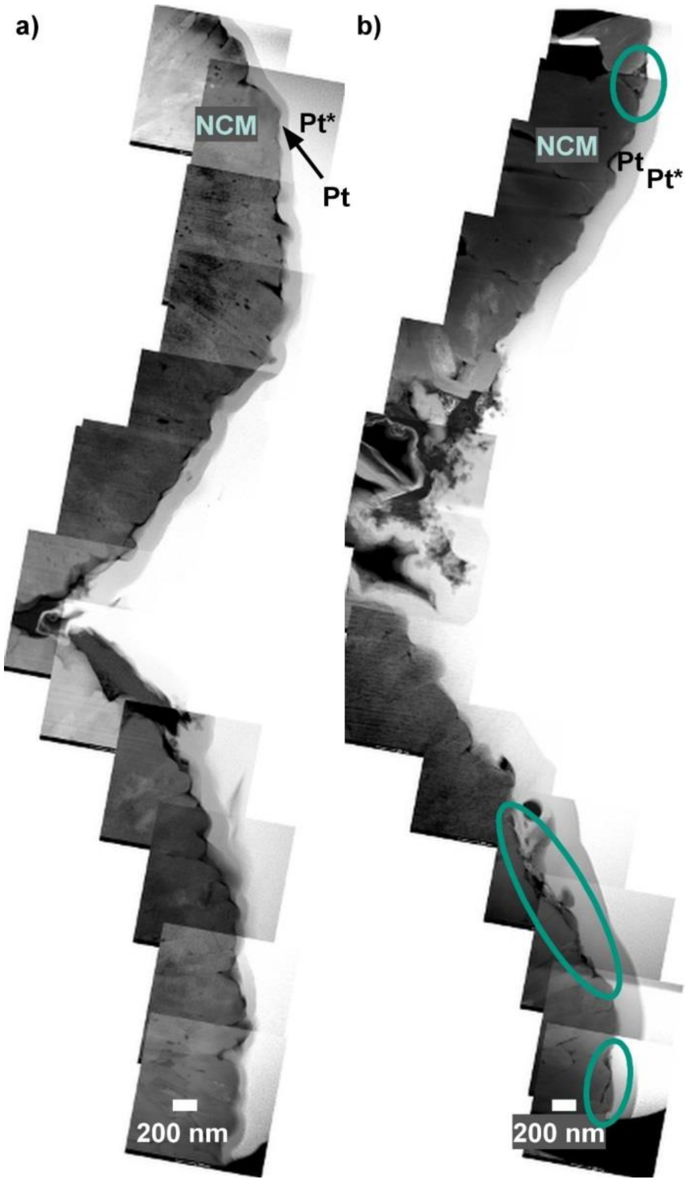

Figure S13. HAADF-STEM images of ALD-10@NCM622 (a) and bare NCM622 (b) after cycling in full-cell configuration for 1400 cycles. The surface of two adjacent secondary particles is reconstructed by stringing together overlapping images. Areas of pronounced surface degradation are denoted by green ellipses. The electrodes were covered by platinum protective layers. The corresponding BF-STEM images are shown in Fig. 6.
